# The Role of Probiotics and Prebiotics in the Prevention and Treatment of Obesity

**DOI:** 10.3390/nu11030635

**Published:** 2019-03-15

**Authors:** Tomás Cerdó, José Antonio García-Santos, Mercedes G. Bermúdez, Cristina Campoy

**Affiliations:** 1 Department of Pediatrics, School of Medicine, University of Granada, Avda. Investigación 11, 18016 Granada, Spain; tcr@ugr.es (T.C.); joseantonio_gsantos@outlook.es (J.A.G.-S.); mgbermudez@ugr.es (M.G.B.); 2 EURISTIKOS Excellence Centre for Paediatric Research, Biomedical Research Centre, University of Granada, 18016 Granada, Spain; 3 Instituto de Investigación Biosanitaria ibs, GRANADA, Health Sciences Technological Park, 18012 Granada, Spain; 4 Spanish Network of Biomedical Research in Epidemiology and Public Health (CIBERESP), Granada’s node, Carlos III Health Institute of Health Carlos III, 28029 Madrid, Spain

**Keywords:** obesity, gut microbiota, probiotics, prebiotics, nutrition

## Abstract

Obesity is a global pandemic complex to treat due to its multifactorial pathogenesis—an unhealthy lifestyle, neuronal and hormonal mechanisms, and genetic and epigenetic factors are involved. Scientific evidence supports the idea that obesity and metabolic consequences are strongly related to changes in both the function and composition of gut microbiota, which exert an essential role in modulating energy metabolism. Modifications of gut microbiota composition have been associated with variations in body weight and body mass index. Lifestyle modifications remain as primary therapy for obesity and related metabolic disorders. New therapeutic strategies to treat/prevent obesity have been proposed, based on pre- and/or probiotic modulation of gut microbiota to mimic that found in healthy non-obese subjects. Based on human and animal studies, this review aimed to discuss mechanisms through which gut microbiota could act as a key modifier of obesity and related metabolic complications. Evidence from animal studies and human clinical trials suggesting potential beneficial effects of prebiotic and various probiotic strains on those physical, biochemical, and metabolic parameters related to obesity is presented. As a conclusion, a deeper knowledge about pre-/probiotic mechanisms of action, in combination with adequately powered, randomized controlled follow-up studies, will facilitate the clinical application and development of personalized healthcare strategies.

## 1. Introduction

The prevalence of obesity has been continuously rising worldwide over the last few decades, such that it is considered a pandemic. According to the World Health Organization (WHO), in 2035, 39% of people in today’s society will be affected by obesity [1]. That is why there is now an increased need to create new public policies in prevention and in primary health care [2].

Obesity is defined as abnormal or excessive fat accumulation that may be harmful to health [3]. Although its etiology has been associated with an energy imbalance between calories consumed and calories expended, mainly as a result of a poor diet and insufficient physical exercise, it is really a compendium of factors, involving an interaction among genetics, hormones, and the environment [4]. In this connection, recent human and animal studies have shown the influence of the gut microbiota community in the development of obesity, regulating host metabolic functions [5,6]. Furthermore, experimental models have shown that several bacterial strains are able to inhibit or attenuate immune responses associated with chronic inflammation [7]. In the meantime, emerging evidence reveals a large number of microorganism genera, such as *Lactobacillus*, *Bifidobacterium*, *Saccharomyces*, *Streptococcus*, and *Enterococcus*, whose supplementation in the diet might play a role in prevention or management of obesity [8]. Several studies have observed that dietary carbohydrates, especially those that are not digested in the upper part of the gut, are able to enhance growth and functions of the gut microbiota community [9,10]. Increasing dietary fiber intake through the use of specific prebiotics may stimulate satiety hormones and enhance appetite control, which may help in body weight control [11].

At present, the question of probiotics and prebiotics influence on host metabolism, and specifically on obesity, is actively debated in the scientific literature because of contradictory data and a lack of knowledge of long-term effects [12,13]. Given the above, the aim of the present review is to outline the main effects of pre- and probiotics in the prevention and treatment of obesity, noting the most recent advances in their implementation in the clinic, as well as the mechanisms of action described so far. To perform the present review of the literature, the PubMed database was explored to acquire updated information; recent knowledge about the different mechanisms of probiotics and prebiotics potentially involved in the prevention and treatment of obesity was considered. The selected papers were those reporting randomized clinical trials as well as cohort and epidemiological studies in humans, mainly published in the last 10 years and reporting associations about the role of probiotics and prebiotics in the metabolic state or obesity treatment. Furthermore, experimental animal studies regarding probiotics and prebiotics for prevention and management of obesity are included here.

## 2. Pathophysiology of Obesity

Obesity is a worldwide epidemiologic syndrome characterized by fat mass accumulation, mainly visceral fat. The prevalence of obesity has doubled in the last three decades; in 2016, according to the WHO, more than 1900 million (39%) people above 18 years old were overweight and 600 million (13%) were classified as obese [14]. Moreover, childhood obesity has grown exponentially over the past 25 years in infants and young children (aged 0–5 years), having increased from 32 million globally in 1990 to 42 million in 2013 and being now the most prevalent nutritional disorder globally among children [15]. Obesity is now declared as a current global epidemic by the WHO. Furthermore, obesity is the common denominator of diet-related chronic diseases such as cardiovascular pathologies or diabetes, but also chronic inflammatory and allergic disorders [15].

Obesity is assessed in practice by body mass index (BMI) or the ratio of waist to hip circumference. High BMI indexes correlate with chronic diseases such as hypertension, dyslipidemia, type 2 diabetes, cardiovascular diseases, and metabolic syndrome, as well as development of some type of cancers [16]. Emerging evidence suggests that dietary habits, high-sugar and saturated fat, also contribute to anxiety and mood disorders, which show bi-directional associations with obesity [17,18].

The pathophysiology of obesity is multifactorial, involving in its development inadequate life style, neuronal and hormonal mechanisms, as well as genetic and epigenetic factors [19], which result from an imbalance between energy intake and expenditure. However, increasing evidence indicates that energy intake and expenditure are highly interconnected and regulated by complex and coordinated mechanisms that ultimately influence hypothalamic, limbic, brain stem, and other central nervous system centers to regulate food intake and energy spending. Metabolic and physiological energy demands and maintenance of ‘‘adequate’’ energy stores serve as positive signals that determine food intake and non-activity-associated energy expenditure. Multiple hormones and neuronal circuits appear to control these regulatory processes, including leptin providing feedback from the fat itself, ghrelin secreted by the gastric mucosa, various intestinal peptides, and several appetite-regulating neuropeptides [20].

Genetic factors clearly contribute to the control of the physiologic response to caloric excess and hence to the development and maintenance of obesity; heritability estimates for the variance in BMI ranging between 40% and 70%. In this regard, specific associations between host genotype and obesity have been revealed. This is the case for leptin and leptin-receptor genes, as well as apolipoprotein A1 and phospholipase D1 encoding genes [16].

However, the dramatic rise in the prevalence of obesity over the past decades has turned attention towards the environment. Greater control of ambient temperature as a socioeconomic development consequence, increased sedentariness or less physical activity as a result of lifestyle changes in Western societies, and the ubiquitous presence of cheap high-calorie foods have all been implicated as important causal factors [21]. In this regard, food products have greatly changed during last decades, taking advantage of diverse food processing and preservation technologies, modifications that have increased intake, beyond that of fresh vegetables and fruits, of higher caloric food with lower nutritional value. The modified fatty acid composition of a Western diet, which is usually rich in saturated and trans-fatty acids, increases the risk of chronic vascular disease by elevating (blood serum) concentrations of total and LDL cholesterol. Moreover, diets high in sodium and low potassium may lead to a variety of chronic illnesses, including hypertension and stroke. Another factor is the presence of dietary fibers, such as inulin, resistant starch, and beta-glucans, which are reduced in Western diet, and are important food components that can delay gastric emptying, reducing appetite and thus helping in the control of caloric intake. Enhanced consumption of high-fat and high-sugar diets have been shown to change microbial ecology, leading to the notion that gut microbiota may function as an *environmental* factor that results in increased energy harvest and obesity [22].

In humans, nutrient digestion and absorption mainly occur in the stomach and proximal small intestine. Carbohydrates are a vital source of energy for the human body, yet humans have very limited abilities to degrade and utilize dietary mono-, oligo-, or poly-saccharides; various members of the gut microbiota, known as saccharolytic microorganisms, degrade these complex glycans thereby providing the host with a variety of metabolites, in particular short-chain fatty acids (SCFAs). In healthy individuals, 66–95% of proteins, 85% of carbohydrates, and 95% of fats are absorbed before entering the large intestine [23]. The highest density of gastrointestinal microorganisms is found in the cecum and proximal colon [24], as the presence of acids in the stomach and bile acids and pancreatic juice in the duodenum and jejunum can inhibit the growth of microorganisms—more so than most bacteria inhabit the large intestine [19].

Human intestinal microbiota represents a complex ecosystem, consisting in numerous diverse sets of microorganisms such as bacteria, viruses, Archaea, fungi, protists, nematodes, as well as phages, deeply implicated in different functions of host metabolism. The gut microbiota is predominantly involved in the fermentation of indigestible carbohydrates into SCFAs, which have been found to exert multiple effects on energy homeostasis and are crucial for intestinal health [22]. The most abundant SCFAs are acetate, butyrate, and propionate, which play an important role as substrates for glucose metabolism; these SCFAs comprise >95% of the SCFA content. Several animal and human studies have found increased SCFA fecal concentrations [in particular propionate] in obese compared to lean individuals, suggesting that increased fecal concentrations of SCFAs are associated with obesity [25,26].

On the other hand, it is well known that obesity is associated with chronic low-grade inflammation and insulin resistance. White adipose tissue is metabolically the most important adipose tissue, playing a central role in the inflammatory state and expressing pro-inflammatory cytokines such as TNF-α and interleukins [IL]-1, IL-6, IL-10, and IL-12 [22]. In obesity, there is increased cytokine production in white adipose tissue and an infiltration of macrophages, which in turn enhances pro-inflammatory cytokines and subsequently induces insulin resistance. A contributing factor to the onset of this chronic low-grade inflammation is thought to be alterations in the composition of the gut microbiota induced by a high-fat diet (HFD). These alterations result in increased gut permeability, otherwise known as gut barrier dysfunction [27]. Gut barrier dysfunction causes low-grade inflammation by either directly translocating Gram-negative intestinal bacteria or increasing lipopolysaccharides (LPSs) [22] originated from the outer membrane of Gram-negative bacteria. that also induces metabolic endotoxemia, which in turn generates low-grade inflammation. LPSs have therefore been postulated to be the molecular link between intestinal microflora and the chronic low-grade inflammation induced by a HFD that leads to insulin resistance. Abnormally increased gut permeability to bacteria and their products is a factor that further contributes to insulin resistance and oxidative stress [21].

In this regard, intestinal microbiota has received increasing attention lately, in particular as a metabolic gateway between the outer environment and the host, regarding the modulation of inflammation, energy metabolism, and body weight homeostasis. It has been shown that obesity may be associated with gut microbiome configuration in humans and that obesity phenotypes can be transmitted via the gut microbiota in rodent models of obesity [28]. Curiously, the microbiome shares properties with both the environment (it is, perforce, an intimate part of the human environment) and genes (it is heritable and contains genetic material). Indeed, some authors have proposed that the microbial genetic material that we carry with us effectively represents an extension of our genome—a “meta-genome” [29]. In this context, alterations in this meta-genome occur on time scales consonant with the observed, rapid increase in obesity prevalence. The gut microbiome thus represents a compelling candidate for being an important contributor to the current increase in obesity rates. Further, accumulating evidence supports a role for the gut microbiome as a modifier of some of the metabolic and end organ complications of obesity.

## 3. The Role of Intestinal Microbiota in the Metabolic State

Obesity and metabolic syndrome in general are influenced by many physiological factors that are strongly associated with diet and lifestyle, in addition to genetic and environmental factors. Diet, clinically defined as the total food intake by an individual over a given time period, is linked to obesity with the gut microbiota also playing an important role [16]. Thus, the hypothesis that obesity can be controlled by modulating the gut microbiota may lead to effective therapeutic interventions.

The gut microbiota (the collective genomic content of microorganisms) in humans contains ~40 trillion microorganisms. The dominating bacteria phyla in humans, accounting for 90% of the gut microbiota, are *Firmicutes* and *Bacteroidetes*. There are currently >274 genera within the *Firmicutes* phylum, including *Bacillus*, *Lactobacillus*, *Mycoplasma*, and *Clostridium*. *Bacteroidetes* includes ~20 genera, of which the most abundant genus in the human gastrointestinal tract is *Bacteroides* [22].

The gut microbiota plays an important role in the absorption, storage, and expenditure of energy obtained from dietary intake [23,24,25,26]. Furthermore, recent animal studies have shown that the gut microbiota is also involved in the regulation of food intake by affecting hormones that influence metabolic function and areas in the brain associated with eating behavior [27]. This so-called “gut microbiota-brain axis” represents a bidirectional signaling axis that regulates body weight by balancing appetite, storage, and energy expenditure [22]. Although reports on the composition of the gut microbiota in obese individuals are not uniform, obese humans showed an increased Firmicutes/Bacteroidetes ratio in the fecal microbiota, and reduced microbial diversity and richness seem to be a recurrent finding. The obese phenotype was transmittable via intestinal microbiota alone in germ-free mice [30] and human beings [31] and was reversed in germ-free mice following co-housing with mice transplanted with the lean microbiota [31]. These findings show the transmissible, rapid, and modifiable nature of interactions between diet and gut microbiota in obesity and metabolic syndrome. Those alterations in diversity and microbial richness are thought to be associated with altered SCFA composition, energy homeostasis, and inflammation. However, the causal relation between gut microbiota composition and energy homeostasis is complex, and contributory variables such as genes, age, and diet substantially affect the function of gut microbiota [32].

Alterations in the composition of the human gut microbiota occur in metabolic disorders such as obesity, diabetes [33], eating disorders, as well as stress-related, neuropsychiatric disorders including depression [34] and anxiety [35]; these pathologies are characterized by changes in eating behavior. In this regard, the gut–brain axis exerts a substantial physiological impact on mood, behavior, and stress responsiveness. Acute and prolonged exposure to stress can change both the quality and quantity of calories consumed, and stress-induced changes in food consumption and energy balance can interact with emotional state [18].

Healthy gut microbiota is crucial for proper metabolic function and homoeostasis, which substantially benefits the host in exchange for living and proliferating in the intestinal habitat. Commensal gut bacteria have a crucial synbiotic relationship with the human body throughout its evolution, protecting and supporting the structure of intestinal mucosa. Gut bacteria are therefore becoming increasingly recognized as key regulators of host physiology and pathophysiology, and undeniably have a role in health and disease [36].

Some mechanisms have been proposed to explain the role of gut microbiota in obesity development. One is related to the energy regulation and ability of the microorganism to ferment dietary polysaccharides not digested by humans [32]. Fermentation of dietary fibers results in SCFA generation. Once absorbed, SCFAs can induce lipogenesis and increase triglyceride stores through molecular pathways. SCFAs have shown to activate the carbohydrate responsive element-binding protein and the sterol regulatory element-binding transcription factor 1, both involved in lipogenesis. Furthermore, SCFAs can suppress the fasting-induced adipocyte factor, which inhibits lipoprotein lipase, inducing triglycerides accumulation in host adipocytes [32]. Obesity-associated microbiota increases the efficiency of calorie uptake from ingested foods and affects energy balance by influencing energy use and storage [20]. Thus, an obesity-associated microbiota provides more energy to the host from otherwise indigestible carbohydrates and proteins than does a lean-associated gut microbiota, via increased production of different primary fermentation enzymes and nutrient transporters [37]. Apart from SCFAs, other bioactive metabolites produced by the intestinal microbiota in a diet-dependent manner are conjugated fatty acids [38], which have peripheral effects and modulate the brain via direct or indirect mechanisms, modifying host metabolism and the central regulation of appetite and food intake [36].

Another mechanism proposed to explain the association between the gut microbiota and obesity is its ability to decrease liver fatty acid oxidation by suppressing the adenosine monophosphate kinase (AMPK). AMPK is found in the liver and in muscle fibers and acts as an indicator of cellular energy; inhibition of AMPK results in decreased fatty acid oxidation and, as a consequence, increased fat accumulation [19].

The gut microbiota changes the composition and relative abundance of bile acid species, which might explain its effect on glucose and insulin homoeostasis [39]. A reduced bile acid concentration in the gut has been associated with bacterial overgrowth and inflammation [39]. Additionally, some gut bacteria metabolize bile acids and their conjugates for a source of energy, causing activation of bile acid receptors essential for maintaining glucose tolerance and insulin sensitivity, in the intestine and liver [36]. Obese patients and those with type 2 diabetes have altered bile acid metabolism [40], and administration of bile acids, in both human and animal studies, led to improved glycemic control. Furthermore, the effect of the gut microbiota on serotonin metabolism might also influence host glucose homoeostasis [36].

Gut microbiota also may contribute to metabolic disturbances observed in obese patients by triggering systemic inflammation [41]. Cell membrane LPSs of those Gram-negative bacteria from gut microbiota bind toll-like receptors (TLRs), mainly TLR4. TLRs are immune transmembrane proteins able to upregulate inflammatory cytokines and chemokines and to engage intracellular signaling pathways, regulating the nature, magnitude, and duration of inflammatory response. Chronic low-grade inflammation appears to be a major factor in the development of obesity-related metabolic disturbances. Studies in mice indicate that an HFD may result in changes in intestinal microbiota composition and increased levels of circulating endotoxins such as LPSs. Dietary fat is crucial in this process because it increases intestinal LPS absorption through incorporation into chylomicrons [36]. Infusion of LPSs causes low-grade chronic inflammation and most of the features of the early onset of metabolic diseases, such as visceral fat deposition, glucose intolerance, and hepatic insulin resistance [21]. Abnormally increased gut permeability to bacteria and their products is a factor that further contributes to insulin resistance and oxidative stress. Consistently, observations in mice indicate that intestinal microbiota influences energy metabolism and has systemic effects on host lipid metabolism, especially triglycerides and phosphatidylcholine. The intestinal microbiota has been shown to metabolize the dietary lipid phosphatidylcholine to trimethyl amine, which promotes atherosclerosis and inflammation in mice [42].

The gut microbiota ecosystem is established after birth following the transfer of maternal and environmental bacteria, and continues to develop until adulthood. After birth, each individual acquires a unique microbiota profile, influenced by various determinants factors, such as the mode of birth delivery, breastfeeding, maternal age and metabolic condition, the use of antibiotics, diet, and urban versus farm living [43]. In this regard, differences in microbiota profile have been reported in babies born through vaginal delivery, with a microbiota more similar to the maternal vaginal one (*Lactobacillus* spp.), and in babies born through Caesarean section that are instead colonized by common skin and environmental microbes (*Staphylococcus*, *Streptococcus*, or Propionibacteria) [44]. Likewise, breastfed and formula-fed infants show differences in microbiota composition: breastfed infants have larger populations of *Lactobacillus* and *Bifidobacterium*, as breastmilk is rich in bioactive ingredients, including human milk oligosaccharides (HMOs), which selectively stimulate their growth. In contrast, formula-fed infant microbiota is characterized by increased bacterial diversity and a high prevalence of *Clostridium difficile*, *Bacteroides*, *Streptococcus*, and *Veillonella* [45,46]. On the other hand, exposition to antibiotics early in life, maternally or via the food chain, can have a large effect on gut microbiota, disturbing its composition and functionality, which in turn can disrupt gut barrier function and lead to influx bacterial fragments into blood. As a result, low-grade chronic inflammation and metabolic endotoxemia are produced, affecting host metabolism and insulin resistance. This microbiota alteration in early life has long-lasting effects on bodyweight in adulthood; epidemiological studies have shown that early exposure to antibiotics is associated with an increased risk of obesity and metabolic disorders later in life [47]). Those microbiota bacteria are important for body homeostasis, by participating in the digestive process, energy regulation, SCFA production, vitamin synthesis, protection against pathogenic microorganisms, and modulation of the immunologic system [48,49]. Alterations in the composition of the microbiota, especially early in life, might cause obesity and diabetes by substantially modifying the host metabolism and affecting homoeostasis and the central appetite mechanism [36]. With respect to this, the best strategy in obesity prevention could be an intervention supporting “healthy” microbiota in order to minimize risk factors presents early in life. Additionally, alterations of the early microbial composition can result in long-term modulation of stress-related physiology and behavior [18]. Moreover, it has been shown that microbial-intestinal epithelial cross-talk regulates cellular function mediated via epigenetic mechanisms [50]; thus, alterations in those epigenetic mechanisms might lead to disease development during life.

Dietary habits are the main contributors to the diversity of the human gut microbiota by altering its composition. Short-term changes in diet profile can shift the gut microbiota. Dietetic intervention could then be a good strategy to treat obesity, by reducing energy intake and potentially modulating gut microbiota to aid weight loss.

## 4. Experimental Studies with Probiotics and Prebiotics for Prevention and Management of Obesity

There is consistent evidence to support obesity as a major public health problem worldwide in all age groups, leading to important efforts aimed at implementing obesity prevention programs and identifying new therapeutic and intervention targets. Classical treatments for obesity include both bariatric surgery and non-surgical multicomponent approaches based on behavior therapy, dietary changes, physical activity, and pharmacotherapies [51]. However, both strategies show side effects and/or high cost that hamper long-term success. In this regard, bariatric surgery has been related to greater body weight loss and control of obesity-related comorbidities, but it is not widely used due to high costs and a high risk of adverse events, including anemia and reoperations. Moreover, this therapeutic strategy is discouraged in the pediatric/adolescent population because, in addition to ethical reasons, its complications and long-term consequences are more severe than they are in the adult population [52,53]. It seems therefore clear that lifestyle interventions should be the main alternative to treat obesity, especially at school-age. In fact, schools are a key place for the initiation of effective anti-obesity policies based on health-promoting environments [54]. Nevertheless, lifestyle changes are also difficult to implement over the long term due to the expectation of tangible results, in terms of weight loss and the modulation of metabolic pathways, in the very short term [55]. As a consequence, research efforts have focused on identifying alternative strategies for long-term prevention and the treatment of obesity. Based on the fact that gut microbiota clearly differs between obese and lean individuals [56], selective modulation of gut microbiota using probiotics and/or prebiotics has emerged as a potential therapy for the control of weight gain in both obese and susceptible-to-obesity subjects [55,56,57]. The discussion below provides experimental data on available probiotics/prebiotics and their anti-obesogenic properties.

According to Food and Agriculture Organization of the United Nations (FAO) and WHO, probiotics are defined as “live microorganisms which, when administered in adequate amounts, confer a health benefit on the host” [58]. Recent systematic review [59] found that specific strains belonging to *Lactobacillus* (*L. casei* strain Shirota (LAB13), *L. gasseri*, *L. rhamnosus*, and *L. plantarum*, among others) and *Bifidobacterium* (mainly *B. infantis*, *B. longum,* and *B. breve* B3) species have been widely used as probiotic treatment in well-established animals models of obesity, due to its lack of pathogenicity and low level of antibiotic resistance. In fact, over 85% of studies reported that both mice and rats fed with the aforementioned strains of *Lactobacillus* and *Bifidobacterium* showed less weight gain, fat accumulation, and white adipose tissue compared to placebo-treated animals. However, experimental studies clearly differ in both treatment duration (ranging from 4 weeks to 6 months) and daily dose administration of probiotics, which lead to higher or lower effects on body weight or fat mass. Interestingly, a detailed analysis of these studies also suggests that tested probiotics exert their anti-obesity effects through species- and strain-specific mechanisms of action, including beneficial changes in gut microbiota, lower insulin resistance, or greater satiety. Other studies using different species and/or strains of *Lactobacillus* and *Bifidobacterium* have failed to demonstrate beneficial effects of probiotic therapy on obese animals. In fact, different strains of *L. plantarum* including *L. plantarum* DSM 15313, *L. plantarum* NCIMB8826, *L. plantarum* strain No 14, as well as *L. acidophilus* NCDC13, *L. gasseri* SBT2025, *L.casei* strain Shirota 4,159,029, and *L. coryniformis* CECT57, had no significant effect on the weight in both obese mice and rats [59]. In Ali et al.’s study [60], administration of a mixture of *L. acidophilus* LA140, *L. casei* LC107, *B. bifidum* BBL730, and isoflavones induced a decrease in body weight and fat accumulation, but this effect was not observed after probiotic supplementation alone. Finally, Bubnov et al. [61] showed that a combination of *B. animalis* VKB and *B. animalis* VKL had no significant anti-obesity effects, although both probiotics administered alone reduced body weight in female BALB/C mice fed with a fat-enriched diet. Thus, both studies seem to suggest that potential interactions between food ingredients and certain probiotic strains should be taken into account in obesity management. Interestingly, there is also some evidence that probiotic supplements based on *L. plantarum* DSM15313 [62], *B. animalis* subsp. lactis BB-12 [63], *Bifidobacterium* M13-4 [64], or a mixture of *B. lactis* Bi1, *B. breve* Bbr8, and *B. breve* [65] led to increased weight gain and body fat. These results may be explained by the fact that some probiotic strains could improve nutrient absorption and gut processes, leading to increased weight gain in the host [66].

In addition to *Lactobacillus* and *Bifidobacterium*, other microorganisms have shown anti-obesogenic effects in animals, including *Pediococcus pentosaceus* LP28, *Bacteroides uniformis* CECT 7771, *Akkermansia muciniphila*, and *Saccharomyces boulardii* Biocodex. In fact, oral administration of *P. pentosaceus* LP28 reduced body weight gain, visceral fat, and liver lipid content in HFD-induced obese mice, suggesting that these anti-obesity effects could be related to the downregulation of genes involved in lipid metabolism [67]. Moreover, *B. uniformis* CECT 7771 [68] and *A. muciniphila*, a mucin-degrading bacterium located in human mucosa [69], have been also identified as beneficial probiotics on the management of obesity-related metabolic and immune dysfunction, including fat-mass gain, metabolic endotoxemia, adipose tissue inflammation, and insulin resistance. Finally, the anti-obesogenic role of probiotic yeast *Saccharomyces boulardii* Biocodex has been established in type-2 diabetic and obese mice [70]. Thus, *Saccharomyces*-treated mice showed lower body weight gain and fat mass compared to untreated mice. Interestingly, these host metabolism responses were linked to major changes in gut microbiota composition, increasing the amount of *Bacteriodetes* while reducing the proportion of phyla related to obesity (*Firmicutes*, *Proteobacteria*, and *Tenericutes*).

In summary, most of the experimental studies support anti-obesity properties of probiotics, mainly *Lactobacillus* and *Bifidobacterium*. However, these studies clearly differ in bacterial strains used, animal obesity models, and treatment period, making it difficult to report the most effective probiotic on weight reduction. Moreover, adverse anti-obesogenic effects of specific probiotic strains were found, reflecting potential practical issues in introducing live microorganisms into the host gut. In this regard, efforts should focus on those key factors in the identification of new probiotic strains as potential obesity therapies, including (i) improvement in strategies for probiotic production, (ii) detailed knowledge of probiotic–host intestinal microbiota interactions, (iii) standardization, as much as possible, of probiotic dosage and treatment duration, (iv) potential effects of physical target subjects (age, gender, and genetic background) on the efficacy of treatment, and (v) the use of effective carriers and functional foods (milk products and soy-based products) in order to improve probiotic’s effects on body weight [71,72].

Prebiotics have been defined by FAO/WHO as “non-digestible food ingredients that beneficially affect the host by selectively stimulating the growth and/or activity of one or a limited number of bacterial species already established in the colon, and thus improve the host health” [72]. According to this concept, prebiotics usually include non-digestible, non-hydrolysable carbohydrate forms (i.e., galacto-oligosaccharides (GOSs), fructo-oligosaccharides (FOSs), soybean oligosaccharides, inulin, ciclodextrins, gluco-oligosaccharides, xylo-oligosaccharides, lactulose, lactosucrose, and isomaltooligosaccharides), with the ability to reach the distal sections of the human gastrointestinal tract where they are used as nutrients by host intestinal bacteria [73].

Experimental studies have shown that the consumption of food rich in prebiotics is strongly related to beneficial effects against obesity, through different mechanisms of action. Among them, there is growing evidence that prebiotic-based therapy changes gut microbiota composition, stimulating the growth of *Lactobacillus* and *Bifidobacterium* in the gastrointestinal tract of obese animals [74] and, at the same time, reducing the population of pathogenic microorganisms including *Firmicutes* and *Bacteroidetes* [75]. Some studies have shown that these changes were related to improved entero-endocrine cell activity, glucose homeostasis, and leptin sensitivity in both obese and diabetic mice treated with oligofructose [76], as well as prebiotic carbohydrates-treated *ob/ob* mice [77]. Interestingly, these changes were also associated with increased endogenous glucagon-like peptide-2 (GLP-2) production, an intestinotrophic pro-glucagon-derived peptide involved in intestinal permeability, thus reducing both obesity-related systemic and hepatic inflammatory disorders.

In addition to modulate gut microbiota composition, anti-obesogenic effects of prebiotics also involve improvement of lipid and glucose metabolism. In this regard, Everard et al. [76] reported that oligofructose-treated animals showed non-obese metabolic phenotypes characterized by lower triglycerides levels, adipose tissue mass, and muscle lipid infiltration. Short chain fructo-oligosaccharides treatment also had beneficial effects on plasma lipid metabolome and insulinemia, which were associated with changes in composition and activity of the intestinal microbiota of diet-induced obese mice [78]. Recently, Nihei et al. [79] found that supplementation with α-cyclodextrins not only modulated intestinal gut microbiota, but also increased lactic acid and SCFAs levels in obese mice. These effects were associated with changes in expression of those genes involved in lipid metabolism, including the upregulation of peroxisome proliferator-activated receptor (PPAR) γ and PPARα, and the downregulation of sterol regulatory element-binding protein-1c (SREBP-1c) and fatty acid synthase, which could partly explain the anti-obesogenic effect of α-cyclodextrins.

As noted above, prebiotic-induced changes in gut microbiota composition lead to improvement in the activity of entero-endocrine cells, which release hormones involved in the modulation of food intake, energy homeostasis, and body weight [80]. As a consequence, anti-obesity properties of prebiotics seem also to be strongly related to the control of satiety hormones. In this regard, Parnell et al. [75] found that obese JCR:La-cp rats fed with a diet rich in prebiotic fiber, including inulin and oligofructose, showed higher circulating Glucagon-like peptide-1 (GLP-1) levels as well as enhanced expression of pro-glucagon and Peptide YY (PYY) genes. However, prebiotic treatment failed to reduce body weight and fat mass, although energy intake was reduced. Another study suggests that prebiotic effects on the control of satiety and food intake are directly attributed to higher SCFA levels, which improve GLP-1, PYY, and ghrelin production and consequently trigger hypothalamic reward mechanisms [77]. Similar results on GLP-1 levels were found using prebiotic- and protein-enriched diets, but no beneficial effects were reported on glucose and lipid profiles [81].

In light of these results, prebiotics should be considered as a potential therapy for the treatment and prevention of obesity. Interestingly, prebiotic food ingredients can be used in combination with probiotic bacteria, which has been termed “synbiotics”, in order to improve their beneficial effects against obesity. For instance, hypercholesterolemic pigs fed with an HFD and treated with synbiotics containing *Lactobacillus acidophilus* ATCC 4962, fructo-oligosaccharide, inulin, and mannitol improved plasma lipid profiles linked to obesity, decreasing plasma total cholesterol, triacylglycerides, and LDL-cholesterol levels [82]. Bomhof et al. [63] also found that synbiotic treatment based on oligofructose and *Bifidobacterium animalis* subsp. lactis BB-12 had major beneficial effects on gut microbiota composition and glycemia in obese rats. Unexpectedly, these effects were lower than prebiotic alone, which also had a major impact on body composition, including reduced energy intake, weight gain, and fat mass. It has recently been suggested that anti-obesogenic outcomes of synbiotics may be dependent on the timing of intervention. In fact, a postnatal diet supplemented with short-chain GOS, long-chain FOS, and *Bifidobacterium breve* M-16V led to optimal early bacterial colonization of gastrointestinal tract with long-term beneficial effects against obesity [83]. Thus, synbiotic-supplemented mice during early life showed a higher *Bifidobacterium* population and less fat accumulation, insulin sensitivity, and dyslipidemia in adult life, thus preventing later obesity and related metabolic disorders. Further experimental studies are still needed to improve knowledge about possible combinations of pre- and probiotics, the timing of intervention, and their potential anti-obesogenic effects; however, their safe use in humans is warranted.

## 5. Review of Clinical Studies Using Probiotics and Prebiotics in Obesity

Due to promising outcomes obtained in experimental studies, both probiotics (Table 1) and prebiotics (Table 2) have been widely tested as a potential obesity therapy in several clinical trials. Focused on a pediatric obese and adolescent population, maternal *Lactobacillus rhamnosus* GG supplementation at 4 weeks before expected delivery and child treatment during the first six months of life determined a healthy growth pattern (lower weight gain) in children at the age of 1 and 4 years, although no evidence of maintenance of the treatment effect was found at 10 years [84]. Interestingly, the use of supplementation based on *L. salivalis* ls-33 [84] or VSL#33 [85] in obese adolescents failed to reduce body weight, waist circumference, and visceral fat. In addition to the absence of recommendations on healthy life habit in both studies, results discussed here suggest that management of obesity based on probiotic intervention should be initiated early in life to avoid obesity and subsequent metabolic consequences in older ages. There is also growing evidence of beneficial effects of *L. rhamnosus* GG treatment on obesity-related non-alcoholic fatty liver disease (NAFLD) in the pediatric population. In fact, Vajro et al. [85] found that high doses of probiotics for 8 weeks decreased hypertransaminasemia in hepatopathic obese children, while a combination of probiotic treatment and lifestyle interventions should be recommended to obtain major effects on BMI and visceral fat. A probiotic supplement called VSL#3 (*Streptococcus thermophilus* DSM24731, *L. acidophilus* DSM24735, *L. delbrueckii* subsp. Bulgaricus DSM24724, *L. paracasei* DSM24733, *L. plantarum* DSM24730, *B. longum* DSM24736, *B. infantis* DSM24737, and *B. breve* DSM24732) has been also tested as therapy in children, showing beneficial effects on BMI, fatty liver, insulin resistance, and GLP-1 levels in treated children [86]. Similar results were found after treatment with a combination of probiotics (*L. acidophilus* ATCC B3208, *L. rhamnosus* DSMZ 21690, *B. lactis* DSMZ 32,296, and *B. bifidum* ATCC SD6576) and healthy lifestyle recommendations [87]. Positive outcomes in reducing body weight were also found after treatment with *B. pseudocatenulatum* CECT 7765 in obese children with insulin resistance [88]. However, the use of supplementation based on *L. salivalis* ls-33 [89] or VSL#33 [90] in obese adolescents failed to reduce body weight, waist circumference, and visceral fat, which could be related to the absence of recommendations on healthy life habits in both studies. The anti-obesogenic role of different strains of *Lactobacillus* and *Bifidobacterium*, alone or in combination, as well as *Pediococcus pentosaceus*, has also been well-established in obese adults, leading to reduced weight gain, BMI, waist circumference, and fat mass [91,92,93,94,95,96].

Although, in general terms, no gender-specific anti-obesogenic effects were found, Sánchez et al. [101] reported that therapy based on *L. rhamnosus* CGMCC1.3724 plus a restricted calorie diet showed markedly higher weight loss in obese women compared to obese men. This gender-specific change would seem to be related to a greater impact on satiety efficiency, eating habits, and mood, which favorably influence obesity management.

Some clinical trials also suggest that the extent of anti-obesogenic effects of probiotics may depend on both the probiotic dose and viable form used. For instance, reduced visceral adipose tissue and waist circumference were only observed after treatment of obese adult with a high dose of *L. gasseri* BNR17 [94]. High- and low-doses of multispecies probiotic Ecologic^®^ (a mixture of different strains of *Lactobacillus* and *Bifidobacterium*) showed similar beneficial effects on body weight, BMI, and fat mass in obese postmenopausal women, but effects on lipid metabolism were significantly higher in those women who received a high-dose supplement [102]. Interestingly, intervention based on *B. animalis* subsp. Lactis CECT 8145, either in viable form or heat-killed cells, had positive but no different effects on anthropometric adiposity biomarkers, including reduced BMI, waist circumference, and waist circumference/height ratio [96].

Clinical results have also found evidence of the effectiveness of probiotic strains on obesity-related metabolic disorders. In this regard, administration of different *Lactobacillus* sub-strains significantly reduced metabolic biomarkers of type 2 diabetes, including fasting plasma and postprandial blood glucose levels, insulin levels, and insulin resistance [21]. Positive results on liver function, glucose metabolism, and pro-inflammatory markers have been found in NAFLD-related obese patients treated with probiotics, including VLS#3 [116], *L. bulgaricus* and *S. thermophilus* [97], and a mixture of *Bifidobacterium*, *Lactobacillus*, *Lactococcus*, and *Propionibacterium* [99]. However, probiotic treatment failed to modulate anthropometric markers in these patients.

Finally, it is also important to highlight that healthy non-obese adults can also benefit from probiotic therapy. In fact, Osterberg et al. [100] reported anti-obesogenic properties of VSL#3 by reducing both body weight and fat accumulation in this population. Similar outcomes were also obtained in adults with obese tendencies who received fermented milk containing *L. gasseri* SBT2055 [98]. In light of these findings, and comparing them to the aforementioned results in obese animal models, the efficacy of probiotic therapy obtained from human studies is still unclear. The lack of consistent results could be due to several factors, including small cohort studies, an absence of long-term follow-up, the use of different probiotic strains, and their variability in action mechanisms. Thus, it seems clear that further studies should be aimed to identify selective probiotic strains that may produce major changes in body weight or fat loss, either alone or in combination with other strains.

Although the beneficial role of prebiotics on obesity has been supported by experimental studies, results obtained from clinical trials are contradictory. Significant decreases in body weight, BMI, and waist circumference have been observed in overweight and obese adults treated (for 12–17 weeks) with yacon syrup [107], oligofructose [110], and rice husk powder/rice bran [106]. However, body weight was not affected by inulin treatment of shorter duration (4–8 weeks), either alone or in combination with FOS [105,117,118]. Long-term treatment for six months based on daily intake of dietary fiber (Litesse^®^ Ultra polydextrose) also failed to modulate body composition in overweight/obese adults, although fat mass, waist circumference, and food intake were markedly reduced using dietary fiber in combination with *B. animalis* subsp. Lactis 420 [113]. Controversial effects of prebiotic therapy on body weight and composition were also found in obese children. In fact, Nicolucci et al. [109] reported a lower body weight, less body fat, and less trunk fat in obese children who received oligofructose-enriched inulin for 16 weeks. These changes in body composition were related to a major modification of gut microbiota composition, which was characterized by an increasing *Bifidobacterium* population. Conversely, oligofructose supplementation combined with healthy lifestyle habits for 12 weeks was not associated with reduced body weight or total fat body [119]. Regarding their role on energy intake, some trials did not support any effect of either long-term prebiotic supplementation (including pre-meal inulin and galacto-oligosaccharides) [120,121] or short-term fructo-oligosaccharides treatment [122], but others reported that dietary intake of oligofructose or inulin for at least two weeks reduced total energy intake in both non-obese and obese adults [103,105,110]. These results seem to suggest that prebiotic supplementation over long periods are needed to obtain beneficial effects on energy intake and consequently on body weight. Overweight and obese children and adults improved satiety cues and reduced prospective food consumption in response to daily supplementation with inulin-type fructans or oligofructose-enriched inulin for 12–16 weeks [108,111]. Interestingly, it has also been reported that daily consumption of diet enriched with oligofructose [79], chicory-derived fructan [104], or FOS [115] for two weeks improved satiety cues in healthy normal weight subjects. Nevertheless, prebiotic effects on satiety were not associated with subsequent weight loss, which may be related to a short duration of treatment. Conversely, research efforts have focused on the role of prebiotics on hormones involved in the body´s energy homeostasis. Clinical evidence showed that circulating levels of peptide YY [104,114], GLP-1 [104], and GLP-2 [112] increased after dietary prebiotic supplementation for two weeks in overweight individuals, but these effects may be partly explained by a high content of non-prebiotic dietary fibers used in dietary interventions. In light of these findings, there is no conclusive evidence supporting dietary prebiotics for obesity management, although their beneficial effects on the regulation of appetite and obesity-related metabolic parameters have been suggested [123]. Moreover, it is also unclear whether prebiotic therapy should be recommended in order to treat obesity-related NAFLD [124].

Thus, further investigations based on well-powered, randomized placebo controlled trials are still needed to implement both pre and/or probiotic treatment as an efficient tool for the prevention and control of obesity and related diseases. Clinical cohorts should consist of a relatively high sample size and should focus on long-term obesity parameters, enabling long-term follow-up studies aimed to develop both clinical and nutritional guidelines for the use of pre- and/or probiotic therapy in obesity management. Prior to these studies, questions about specific bacterial strains, dose, and the duration of treatment still need to be answered. Despite these shortcomings, this type of therapy has emerged as a unique and exciting opportunity in the management and prevention of obesity and its associated metabolic consequences.

## 6. Probiotics Mechanisms of Action

Modern *-omics* and improved next-generation sequencing techniques support the idea that gut microbiota may exert effects not only locally within the intestine, but also conferring systemic effects and profoundly influencing host metabolism. Therefore, knowing the mechanisms of action of probiotics on host metabolism will allow us to modulate the intestinal microbial community in order to reduce the susceptibility to obesity, as well as other implications for the clinical practices in pediatric endocrinology, gastroenterology, and nutrition. Probiotic organisms are crucial for the maintenance of balance in human intestinal microbiota. Numerous scientific reports confirm their positive effect in the host’s health. Probiotic microorganisms are attributed a high therapeutic potential in obesity, insulin resistance syndrome, type 2 diabetes, and other pathologies [56].

**Figure 1:**
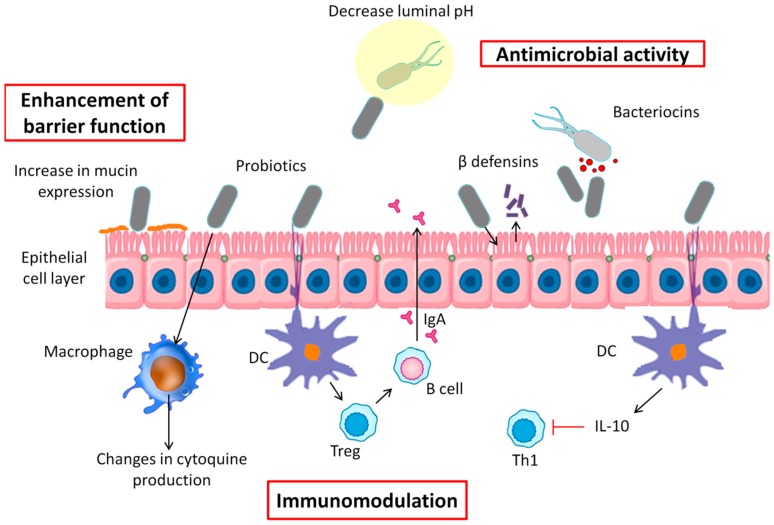
A schematic diagram about potential mechanisms whereby probiotic bacteria might perform within the intestine. These mechanisms include antagonistic effects on various microorganisms, competitive adherence to the mucosa and epithelium (antimicrobial activity), increased mucus production and enhanced barrier integrity (enhancement of barrier function), and modulation of the human immune system (immunomodulation).

It is well known that probiotics have multiple and diverse influences on the host in different ways (Figure 1): antagonistic effects on various microorganisms and competitive adherence to the mucosa and epithelium (antimicrobial activity), increased mucus production and enhanced barrier integrity (enhancement of barrier function), and modulation of the human immune system (immunomodulation) [125]. All of these mechanisms are routed affecting the development of a microbiota, inhabiting the host in a way that ensure a proper balance between pathogens and the microorganisms needed for the optimal function of the host [56].

### 6.1. Antimicrobial Activity

Probiotic activity can fight pathogenic bacteria by decreasing luminal pH, blocking bacterial adherence and translocation, or secreting antibacterial substances and defensins [126]. To resist the colonization of pathogenic bacteria, probiotics are able to alter the environment, making it physiologically restrictive by hydrogen sulfide production and pH/redox potential alterations. For example, it has been reported that *Bacteroides* spp. present sensitivity to mildly acidic pH; by contrast, *Firmicutes* spp. and *Bifidobacteria* are less affected by a decrease in pH, being more tolerant to acid environments [127]. In this connection, Yang et al. observed that, in piglets, supplementation with *Lactobacilli* resulted in a decrease of the colonic luminal pH due to the production of lactic acid, which affected the composition of the microbiota, especially pathogenic bacteria [128]. Furthermore, several studies have observed that a high degree of carbohydrate fermentation, leading to SCFAs, lowers the environmental pH of the colon, promoting the growth of butyrate producers, such as *Roseburia intestinalis*, *Eubacterium rectale*, and *F. prausnitzii*, and inhibiting overgrowth of pH-sensitive pathogenic bacteria [129,130].

Apart from pH alteration, many bacteria can produce antimicrobial peptides, such as bacteriocins, which can be classified by method of killing, genetics, molecular weight and chemistry and method of production [131]. Several studies have described strains of *Lactobacilli*, producers of bacteriocins, whose genomes are associated with weight gain or weight loss [132,133,134]. It should be noted that more bacteriocins were encoded in weight-gain-associated genomes than weight-loss-associated genomes [135]. On the other hand, it has been observed that the *L. reuteri* strain ATCC 55,730 is able to produce a broad-spectrum antibiotic reuterin, named 3-hydroxypropionaldehyde, which, besides acting against Gram-positive and Gram-negative bacteria, act against fungi, protozoa, and viruses [136].

Other antimicrobial peptides are defensins, which are involved in innate defense mechanisms. Probiotic strains can provoke the release of defensins from epithelial cells, stabilizing gut barrier function and acting against pathogens [137]. Wehkamp et al. showed that *E. coli* Nissle 1917 induces human β-defensin-2 gene expression in the Caco-2 intestinal epithelial cell line through NF-kB and AP-1 signaling pathways [138]. Similar results were found using others strains, including *L. acidophilus, L. fermentum*, *L. paracasei* subsp. paracasei, *Pediococcus pentosaceus*, and the probiotic formula VSL#3 [139]. Moreover, some probiotic microorganisms are natural producers of group B vitamins, and products of their metabolism may also show antibiotic, anticancerogenic, and immunosuppressive properties [51], which could contribute to the maintenance of beneficial gut bacteria.

### 6.2. Enhancement of Barrier Function

In the intestine, only one layer made up of epithelia cells conform a physical barrier between the intestinal lumen, the lamina propria, and the mucosal-associated lymphoid tissue. Furthermore, goblet cells, which are simple columnar epithelial cells, secret a mucus able to separate the bacteria from the lumen, preventing colonization of the epithelium [140]. Therefore, epithelial barrier disruption will lead to different illnesses, such as celiac disease [141], inflammatory bowel disease [142], autoimmune diseases (e.g., type 1 diabetes) [143], or enteric infections [144]. As mentioned earlier in this review, abnormally increased gut permeability to bacteria and their products is a factor that further contributes to insulin resistance, oxidative stress, and a level of chronic low-grade inflammation, which, in turn, are associated with the development of obesity-related metabolic disturbances.

The use of probiotics may help to prevent dysbiosis, helping to restore the barrier function through the modulation of cytoskeletal and tight junctional protein phosphorylation or promoting mucus secretion. Guo et al. showed that *Bifidobacterium infantis* and *Lactobacillus acidophilus* protected the intestinal barrier against IL-1β stimulation by normalizing the protein expression of occludin and claudin-1 and by preventing IL-1β-induced NF-κB activation in Caco-2 cells, which may be partly responsible for the preservation of intestinal permeability [145]. Furthermore, it has been reported that several *Lactobacillus* species can block pathogenic *E. coli* invasion and its adhesion, increasing mucin expression in Caco-2 (MUC2) and HT29 (MUC2 and 3) human intestinal cell lines [146,147]. In mouse experimental models, Urdaci et al. observed that the use of *Bacillus subtilis* CU1 and *L. plantarum* CNCM I-4547 showed an impact on diarrhea through limitation of water excretion, involving paracellular permeability or electrolyte transport for those probiotics, respectively [148]. Additionally, Resta-Lenert et al. observed that the administration of *S. thermophilus* and *Lactobacillus acidophilus* maintained or enhanced cytoskeletal and tight junction protein structures in epithelial cell lines exposed to *Escherichia coli* EIEC 029:NM [149].

### 6.3. Immunomodulation

In recent years, researchers have found a close link between gut microbiota and the immune system, where probiotics are able to exert control over epithelial cells, dendritic cells (DCs), monocytes, macrophages, and lymphocytes through different mechanisms. A possible pathway may be the form in which epithelial cells can perceive and distinguish what it is a commensal or a pathogenic bacteria, through cytokine production and signal transduction. Otte et al. observed that *E. coli* Nissle 1917 and VSL#3 were able to affect the regulation of trans-epithelial electrical resistance (TEER) in T84 and HT-29 cells [150]. Moreover, how the epithelial barrier function, after being reduced by pro-inflammatory cytokines, is restored by *Lactobacillus rhamnosus* GG via repair of the TEER level has been described [151]. In addition, in vivo studies of rodents fed dextran sulfate sodium (DSS) have shown that using single strains of probiotics such as *L. brevis*, *L. plantarum*, *L. casei*, and *B. infantis* were able to prevent acute and chronic colitis [152,153,154].

In the intestine, DCs contribute to oral tolerance, producing IL-10 and TGF, which induce regulatory T cells and IgA-producing B cells [155]. Moreover, intestinal DCs are to blame for interacting with luminal bacteria through epithelial tight junctions and with bacteria that have gained access via M-cells [156,157]. Accordingly, several studies have focused on studying the effects of probiotic bacteria on DCs. Hart et al. found that the use of VSL#3 is able to induce IL-10 by DCs from blood and intestinal tissue, and to inhibit the generation of Th1 cells [158]. A recent study has shown that *Lactobacillus bulgaricus* inhibited the local transcription of asthma-associated genes such as GATA3 and STAT6, and increased the expression of T-bet cell specific transcription factor. Furthermore, Kalinina et al. observed in vitro and in vivo that exopolysaccharide secreted by a commensal bacterium, *B. subtilis*, can generate inhibitory DCs [159].

Following DCs, blood monocytes and tissue macrophages are both the second most effective presenters of antigens to memory T cells [160]. It has been observed that *L. casei* strain Shirota has the ability to modify IL-12 and IL-10 production by macrophages through an increase in ligands for TLR3 and TLR5 and ligands for TLR2, TLR4, TLR7, and TLR9, respectively [161]; these results show that probiotic induction of IL-10 and IL-12 production can be flexibly modified by co-stimulation with microbial components; thereby, probiotics may be applied as immunomodulators.

Regarding lymphocytes and its relation with probiotic effects, their clinical significance has been discussed in various diseases. In treating infantile colic, it has been reported that *L. reuteri* DSM17938 enhanced the expression of FOXP3, a master regulator in the development and function of regulatory T cells, also decreasing fecal calprotectin [162], a marker of inflammatory bowel conditions. Lee et al. studied, in nondiabetic participants, the impact of daily consumption of *Weissella cibaria* JW15 on natural killer (NK) cells, observing an increase of NK cell activities and a decrease of IFN-γ levels. Thus, the authors suggested that this probiotic effectively enhanced immune functions in healthy subjects [163].

Preservation of a balanced immune response is thus crucial for the host, as chronic low-grade inflammation and insulin resistance are characteristic of obesity. As mentioned earlier in this review, a contributing factor to the onset of chronic low-grade inflammation is thought to be alterations in the composition of gut microbiota induced by an HFD. Changes in diet, from a Mediterranean to a Western diet with high content in sugars and saturated fats, are driving to obesity via different mechanisms in which changes in gut microbiota play a key role. On the other hand, different bacterial strains have shown beneficial anti-obesity effects, such as a reduction in tissue inflammation, endotoxemia, adiposity, body weight, leptin levels, and energy intake [36]. Thus, dietary intervention by probiotic administration might be one of the approaches by which a “healthy” microbiota can be modulated and maintained.

Nevertheless, as stated in this review, only a small proportion of probiotics have been evaluated regarding their effects in obesity management. Conclusions drawn from the different studies presented in this review must be evaluated with caution due to the presence of concerns regarding methods used to evaluate probiotic impact on animal body weight, which significantly varied from study to study, as well as the dose of probiotics and the duration of administration [71]. Thus, a deep understanding of the potential use of Gram-negative and anaerobic bacteria, parasites, and other microorganisms alone or in combination as potential probiotics, as well as the interactions between them and diet, is still a long way ahead.

## 7. Prebiotics: Mechanism of Action

The gut microbiota community presents an extensive genetic potential involved in many metabolic functions, whose modulation may improve the health of the host. This modulation can be achieved through the use of prebiotics, which are short-chain carbohydrates with a degree of polymerization of between two and about sixty and are non-digestible by human or animal digestive enzymes [164]. Since prebiotics are not the only substances with an ability to alter the intestinal environment, the capacity of selective utilization differentiates prebiotics from other undigested dietary ingredients and compounds, such as antibiotics, minerals, and vitamins [165].

The presence of prebiotics in the diet usually found in fruits and vegetables may lead to numerous health benefits. Among the advantages of those prebiotics, the reduction of the blood low-density lipoprotein level, the stimulation of the immunological system, the increased absorbability of calcium, the maintenance of correct intestinal pH value, and the low caloric value, among others, are worth mentioning [56].

**Figure 2:**
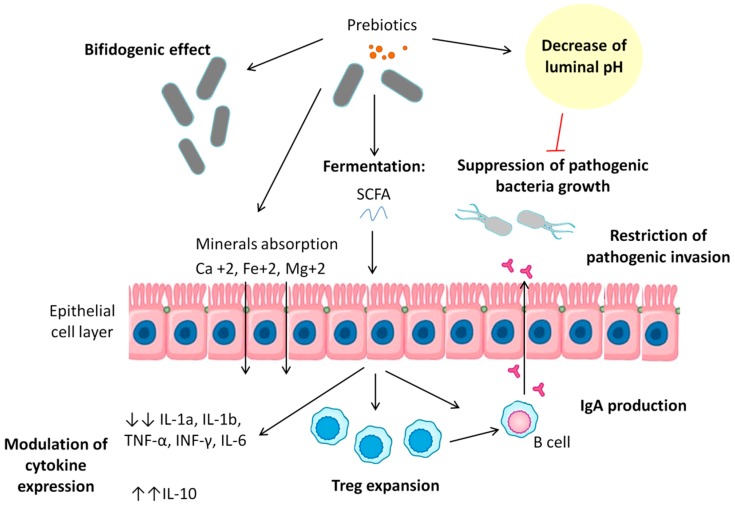
Mechanism of prebiotic action. These mechanisms include the production of microbial metabolic products, noting short-chain fatty acids (SCFAs), the promotion of ion and trace element absorption (such as that of calcium, iron, and magnesium), a decrease in luminal pH, and the regulation of the immune system (increasing IgA production and modulating cytokine production).

Recent studies have suggested that mechanisms through which prebiotics confer benefits to the host (Figure 2) are mediated by microbial metabolic products, noting SCFAs, the promotion of ion and trace element absorption, such as that of calcium, iron, and magnesium, and the regulation of the immune system, increasing IgA production and modulating cytokine production [166].

Prebiotics have a bifidogenic effect, providing a fermentable food source that allows for the increase in the growth of specific beneficial microbial populations such as *Lactobacilli* and *Bifidobacteria* [167]. This can be seen, comparing different types of infant feeding, where those fed with formulas supplemented with GOS and FOS showed an increase in levels of *Bifidobacterium* compared to those who were fed with formula not supplemented with prebiotics [168]. In addition, prebiotics can also restrict invasion of pathogenic bacteria, as noted in the anti-adhesive properties of milk fat globule membrane (MFGM) against enteropathogenic bacteria and enterotoxins [169].

On the other hand, prebiotics are able to improve the absorption of certain ion and trace elements. Connie et al. studied the potential effect of GOS for improving mineral balance and bone properties in mice [170]. Dietary GOS significantly decreased cecal pH and increased net magnesium absorption, calcium, and magnesium retention, improving femur and tibia breaking strength. In addition, Sazawa et al. studied the impact of milk fortified with prebiotics and probiotics on the prevention of diarrhea and on iron status in children at 1–3 years old; they showed that milk supplemented with *B. lactis* and GOS reduced the proportion of iron-deficient children by 35% compared with the control group [171]. However, it was unclear if the upgrade was due to iron absorption enhancement or due to the reduction of bloody diarrhea through the restoration of gut microbiota balance.

Although the conventional dogma is that iron is absorbed predominantly in the duodenum, prebiotic fermentation can decrease pH in the colon, promoting the reduction of Fe (III) to Fe (II) and thus favoring iron absorption [172]. Through fermentation, probiotic bacteria use prebiotic fibers, such as a carbon source, to generate large amounts of SCFAs (lactate, pyruvate, and acetate), which are used by other colon bacteria as starting units for propionate and butyrate production [173]. Butyrate is considered one of the most important colon metabolites due to its anti-inflammatory properties, including the promotion of the expansion of regulatory T cells via the inhibition of histone deacetylation and the induction of IgA production by mucosal B cells [174,175].

Several studies have discussed potential prebiotic effects on the modulation of cytokine expression. Cani et al. observed in obese mice fed with prebiotic carbohydrates a lower profile of plasma LPS, a large variety of pro-inflammatory cytokines, such as IL-1a, IL-1b, TNF-α, INF-γ, and IL-6, and a reduced hepatic expression of inflammatory and oxidative stress markers [77], which has been confirmed in other studies [176]. Moreover, Vulevic et al. found, in healthy elderly volunteers fed with a mixture of GOS, an increase in the production of anti-inflammatory cytokine (IL-10) and a reduction in pro-inflammatory cytokines (IL-1, IL-6, and TNF-α) production [177]. In other human studies, Dehghan et al. detected a significant decrease in levels of IL-6, TNF-α, and plasma LPSs when supplementation with oligofructose-enriched inulin was compared with maltodextrin [178].

However, despite the number of human and in vitro/animal studies aiming to elucidate the potential mechanisms of prebiotics, their long-term effects in host health are still unclear, especially in early life (fetal and neonatal period), infants, and young children; thus, more studies are needed to clarify their mechanisms and effects on health.

Prebiotics may be used as an alternative to probiotics or as an additional support for them [56]. The development of bio-therapeutic formulas containing both appropriate microbial strains and synergistic prebiotics may lead to the enhancement of probiotic effects in the small intestine and the colon. Such “enhanced” probiotic products may be even more effective, and their protective and stimulatory effects may be superior to their components administered separately. Further studies on the combinations of probiotics and prebiotics, as well as the development of synbiotics, could explain the mechanisms of actions of these components, which might confer a beneficial effect on human health.

## 8. Conclusions and Perspectives

The prevalence of obesity is rising throughout the world, reaching pandemic proportions and having major health and economic impacts on society at large. Obesity is a consequence of energy disbalance, involving other factors such as inadequate lifestyle, brain function, and hormonal mechanisms, as well as genetic and epigenetic factors. This multifactorial pathogenesis may in part explain that clinical treatment of obesity represents an important health policy challenge. While bariatric surgery showed beneficial effects, reducing body weight and controlling obesity-related comorbidities, this procedure is highly invasive. There is a high risk of adverse events, and they are safety issues in pediatric populations. Thus, it seems clear that major obesity treatment should be based on a multicomponent approach involving behavior therapy, dietary changes, physical activity, and pharmacotherapies. However, obese subjects expect tangible results in the very short term, making the long-term implementation of lifestyle changes difficult.

Gut microbiota plays a key role as a modulator of energy homeostasis and fat deposition, acting as a connection between host and environmental factors. Composition of the gut microbiota in obese subjects differs from that in lean individuals, and the association of dysbiosis with obesity and related metabolic problems has been shown both in animals and humans. However, which gut microbiota components are the cause of weight gain and abnormal glucose and fat metabolism, and which are protective against obesity and metabolic derangement, is still under investigation. Several studies have shown potential therapeutic effects of probiotics and/or prebiotics on body weight, BMI, waist circumference, fat deposition, lipid profile, and chronic inflammation state, which may lead to new approaches in the treatment and prevention of obesity and related metabolic disorders. Interestingly, a strain-specific effect on body weight and metabolism of the probiotic has also been reported; nonetheless, identification of strains potentially associated with a beneficial effect is lacking, so their systematic use cannot yet be recommended in obesity treatment and associated metabolic disturbances. The dosage, duration of treatment, and long-term effects of the administration of the different strains are still a matter of research; more studies are needed before probiotics can be rationally prescribed for the prevention or treatment of obesity. Control of the diet as well as environmental and lifestyle factors that favor obesity development remain the best solution to problems related to weight gain. Further investigations, including well powered, randomized, and controlled clinical trials, are needed to better understand the mechanisms involved in the anti-obesogenic effects of pre- and/or probiotics in order to develop safe strategies in the prevention and management of obesity.

## Figures and Tables

**Table 1:** Summary of anti-obesity effects of probiotics reported in randomized controlled clinical trials.

Author/Year	Study Design	Population Characteristics	Intervention	Control/Placebo Group	Duration	Clinical Findings (vs. Control/Placebo Group)
Alisi et al. (2014) [86]	Parallel-arm, double-blind RCT	Children aged 11 years with NAFLD (n = 44); intervention (n = 22); placebo (n = 22)	VSL#3 (450 billion bacteria per sachet, one sachet/day) + low calorie diet + moderate physical activity	1 cap/day + healthy habits (low calorie diet + moderate physical activity)	4 months	<BMI, fatty liver, insulin resistance; >GLP-1
Aller et al. (2011) [97]	Randomized, double-blind, parallel, placebo-controlled trial	Patients with NAFLD (n = 28)	500 million of *Lactobacillus bulgaricus* and *Streptococcus thermophilus* (1 tablet/day)	1 cap/day of starch	3 months	Improved liver function, glucose metabolism and pro-inflammatory markers; no changes in anthropometric measures
Famouri et al. (2017) [87]	Triple-blind randomized placebo-controlled clinical trial	Obese children and adolescents (12.7 years) with NAFLD (n = 64); intervention (n = 32); placebo (n = 32)	*L. acidophilus* ATCC B3208 (3 × 10^9^ CFU), *L. rhamnosus* DSMZ21690 (6 × 10^9^ CFU), *B. lactis* DSMZ 32,296 (2 × 10^9^ CFU) *and B. bifidum* ATCC SD6576 (2 × 10^9^ CFU) cap/day + healthy lifestyle habits	1 cap/day of placebo + healthy lifestyle habits	12 weeks	=BMI, weight; <WC
Gomes et al. (2017) [92]	Randomized, double-blind, placebo-controlled, two arm, parallel-group clinical trial	Obese women aged 20–59 years (n = 43); intervention (n = 21); placebo (n = 22)	*L. acidophilus* LA-14, *L. casei* LC-11, *Lactococcuslactis* LL-23, *B. bifidum* BB-06, *B. lactis* BL-4 (2 × 10^10^ CFU/day) + dietary intervention	1 cap/day placebo + dietary prescription	8 weeks	=BMI and weight; <WC
Higashikawa et al. (2016) [93]	Randomized, double-blind, placebo-controlled clinical trial	Overweight adults aged 20–70 years (n = 62); Intervention I (n = 21); Intervention II (n = 21); placebo (n = 20)	Intervention I: Living LP28; Intervention II Heat-killed LP28 (*Pediococcus pentosaceus*) (10^11^ CFU/day)	1 cap/day placebo	12 weeks	<BMI, WC after Intervention II
Jung et al. (2015) [91]	Double-blind, placebo-controlled, randomized clinical trial	Obese adults aged 20–65 years (n = 120); intervention (n = 60); placebo (n = 60)	*L.curvatus* HY7601 + *L. plantarum* KY1032 (2.5 × 10^9^ CFU of probiotics/2 cap/day) + healthy lifestyle habits	2 cap/day placebo + healthy lifestyle habits	12 weeks	<Body weight, WC and fat
Kadooka et al. (2010) [98]	Multicenter, double-blind, randomized, placebo-controlled intervention trial	Adults aged 33-63 years with obese tendencies (n = 87); intervention (n = 43); control group (n = 44)	Fermented milk containing *Lactobacillus gasseri* SBT2055 (5 × 10^10^ CFU/100 g fermented milk). Intake of 200 g/day	Intake of 200 g/day of fermented milk without probiotic	12 weeks	<Abdominal visceral, subcutaneous fat areas, body weight and BMI
Kim et al. (2018) [94]	Randomized, double-blind, placebo-controlled trial	Obese adults aged 20–75 years (n = 90); low-dose intervention (n = 30); high-dose intervention (n = 30); placebo (n = 30)	Low (10^9^ CFU/day) and high (10^10^ CFU/2 cap/twice a day) dose of *Lactobacillus gasseri* BNR17 + lifestyle changes	2 cap/twice a day of placebo + lifestyle changes	12 weeks	<Visceral adipose tissue; WC in high-dose group; <WC in low-dose group
Luoto et al. (2010) [84]	Randomized, double-blind, prospective follow-up study	Mother–child pairs (n = 113); intervention (n = 54); placebo (n = 59)	*Lactobacillus rhamnosus GG* (1 × 10^10^ CFU/day)	1 cap/day of placebo (microcrystalline cellulose)	Mothers 4 weeks before expected delivery; in infants up to 6 month old	<Weight gain at 1 year of life and 4 years; no changes in later stages of development
Minami et al. (2018) [95]	Randomized, double-blind, placebo-controlled trial	Healthy pre-obese adults aged 20–64 years (n = 80); intervention (n = 40); placebo (n = 40)	*Bifidobacterium breve* B-3 (10^10^ CFU/2 cap/day)	2 cap/day of placebo	12 weeks	<Body fat mass
Mykhal´chyshyn et al. (2013) [99]	Open label study	Adult patients with T2D and NAFLD (n = 72); intervention (n = 45); control group (n = 27)	“Symbiter” containing concentrated biomass of 14 alive probiotic bacteria + oral antidiabetic therapy	Only hypoglycemic drugs	4 weeks	<Pro-inflammatory markers; no changes in anthropometric measures
Osterberg et al. (2015) [100]	Randomized, double-blind placebo-controlled clinical trial	Healthy non-obese young male adults (18–30 years) (n = 20); intervention (n = 9); placebo (n = 11)	Two sachets of VSL#3 (450 billion bacteria per sachet in milk shake/once a day) + high fat diet (HFD)	Two sachets of placebo in milk shake/once a day + HFD	4 weeks	<Weight and fat
Pedret et al. (2018) [96]	Randomized, parallel, double-blind, placebo-controlled trial	Abdominally obese adults (n = 126); Intervention I (n = 42); Intervention II (n = 44); placebo (n = 40)	*Bifidobacterium animalis* subsp. Lactis CECT 8145 (Intervention I) or its heat-killed form (Intervention II) (10^10^ CFU/cap/day)	1 cap/day of placebo	3 months	<BMI, WC and waist circumference/height ratio; no differences between live and heat-killed form
Sánchez et al. (2017) [101]	Double-blind, randomized, placebo-controlled trial	Obese adults aged 18–55 years (n = 125); intervention (n = 62); placebo (n = 63)	*L. rhamnosus* CGMCC1.3724 (1.62 × 10^8^ CFU/2 cap/day) + healthy eating behavior	250 mg of maltodextrin + 3 mg magnesium stearate + healthy eating behavior	12 weeks	<Weight
Sanchis-Chordá et al. (2018) [88]	Double-blind, randomized, placebo-controlled trial	Obese children (aged 10–15 years) with insulin resistance (n = 48); intervention (n = 23); placebo (n = 25)	*B. pseudocatenulatum* CECT 7765 (10^9−10^ CFU/day) + dietary recommendations	Placebo + dietary recommendations	13 weeks	<Weight body
Szulinska et al. (2018) [102]	Randomized-double-blind, placebo-controlled clinical trial	Obese postmenopausal women aged 45–70 years (n = 81); low-dose intervention (n = 27); high-dose intervention (n = 27); placebo (n = 27)	Low (2.5 × 10^9^ CFU/day) and high dose (10^10^ CFU/day/two sachets per day) of probiotic mixture including nine different strains of *Lactobacillus and Bifidobacterium*	1 cap/day of placebo	12 weeks	<Body weight, BMI and fat mass in low and high-dose group; improved lipid metabolism in the high-dose group
Vajro et al. (2011) [85]	Double-blind, placebo-controlled pilot study	Obese children (aged 10–13 years) with hypertransaminasemia and ultrasonographic bright liver (n = 20); intervention (n = 10); placebo (n = 10)	*Lactobacillus rhamnosus GG* (12 billion CFU/day)	1 cap/day of placebo	8 weeks	<Hypertransaminasemia Effects on BMI and visceral fat in combination with lifestyle interventions

BMI: body mass index; CFU: colony-forming units; GLP-1: glucagon-like peptide-1; NAFLD: non-alcoholic fatty liver disease; T2D: type 2 diabetes; WC: waist circumference.

**Table 2:** Summary from clinical studies of impact prebiotic on obesity and associated diseases.

Author/Year	Study Design	Population Characteristics	Intervention	Control/Placebo Group	Duration	Clinical Findings (vs. Control/Placebo Group)
Cani et al. (2006) [103]	Single-blinded, cross-over, placebo-controlled design, pilot study	Healthy non-obese adults aged 21–35 years (n = 10); intervention (n = 5); placebo (n = 5)	Prebiotic-supplemented diet (16 g oligofructose/day) divided into breakfast (8 g) and dinner (8 g)	Placebo (dextrin maltose) (16 g/day) divided into breakfast (8 g) and dinner (8 g)	2 weeks	>Satiety; <hunger, energy intake after dinner and total energy intake
Cani et al. (2009) [104]	Randomized, double-blind, parallel, placebo-controlled trial	Healthy non-obese adults aged 21–38 years (n = 10); intervention (n = 5); placebo (n = 5)	Prebiotic-supplemented diet (16 g chicory-derived fructan/day) divided into breakfast (8 g) and dinner (8 g)	Placebo (dextrin maltose) (16 g/day) divided into breakfast (8 g) and dinner (8 g)	2 weeks	>GLP-1, PYY <Hunger No effects on satiety
Dehghan et al. (2014) [105]	Triple-blind randomized controlled study	Adult women with T2D aged 20–65 years (n = 49); intervention (n = 24); placebo (n = 25)	Prebiotic-supplemented diet (10 g inulin/day)	10 g maltodextrin/day	8 weeks	<Fasting glucose, energy intake and pro-inflammatory and oxidative markers
Edrisi et al. (2018) [106]	RCT	Overweight and obese adults (n = 105) aged 20–50 years; Intervention I (n = 35); Intervention II (n = 35); control (n = 35)	Energy-restricted diet containing rice bran (Intervention I) or rice husk powder (Intervention II) (according to DRIs)	Low-calorie diet	12 weeks	<Weight, BMI, WC and pro-inflammatory markers
Genta et al. (2009) [107]	Double-blind, placebo-controlled study	Obese women aged 31–49 years (n = 35)	Yacon syrup (approximately 12.5 g FOS/day) + healthy hypocaloric diet	Placebo syrup (tartaric acid 2.5%, carboxymethylcellulose 1.8%, saccharine 2.5% and glycerine 10%) + healthy hypocaloric diet	17 weeks	<Body weight, BMI, WC, fasting serum insulin, HOMA; >satiety; no changes in total cholesterol and triglycerides
Hume et al. (2017) [108]	Randomized, double-blind, placebo-controlled trial	Overweight and obese children aged 7–12 years (n = 42); intervention (n = 22); control (n = 20)	8 g oligofructose-enriched inulin/day	Equicaloric dose of a 3.3 g maltodextrin placebo/day	16 weeks	>Satiety, prospective food consumption and ghrelin. <Energy intake
Nicolucci et al. (2017) [109]	Single center, double-blind, placebo-controlled trial	Overweight or obesity children aged 7–12 years (n = 42); intervention (n = 22); control (n = 20)	8 g/day (13.2 kcal/day) of oligofructose-enriched inulin	Equicaloric dose of a 3.3 g maltodextrin placebo/day	16 weeks	<Body weight z-score, percent body fat and trunk fat. >*Bifidobacterium* <Bacteroides
Parnell et al. (2009) [110]	Randomized, double-blind, placebo-controlled trial	Overweight and obese adults aged 20–70 years (n = 39); intervention (n = 21); control (n = 18)	Prebiotic-enriched diet (21 g oligofructose/day)	Equicaloric amount of maltodextrin placebo	12 weeks	< Body weight, fat mass, energy intake, postprandial ghrelin and insulin; no effects on postprandial glucose, PYY and GLP-1
Reimer et al. (2017) [111]	Single-centre, placebo-controlled, double-blind RCT	Adults with overweight/obesity aged 18–75 years (n = 96); control (n = 27); prebiotic (n = 26); protein bar (n = 21); combination (n = 22)	(1) control bar; (2) prebiotic bar (inulin-type fructans with 6 g oligofructose + 2 g inulin from chicory root); (3) protein bar (5 g whey protein); (4) combination bar (8 g inulin-type fructans + 5 g whey protein).	Control isocaloric bar (100 kcal/bar)	12 weeks	<Body fat in (3) <Hunger, desire to eat and prospective food consumption in (2), (3) and (4) *>Bifidobacterium* in (2) and (4)
Russo et al. (2012) [112]	Cross-over RCT, double-blind	Healthy males adults aged 18–20 years (n = 20); intervention (n = 10); control (n = 10)	Prebiotic-supplemented diet (11% inulin-enriched pasta)	Control pasta diet (100% durum wheat semolina)	5 weeks	>Neurotensin, somatostatin, GLP-2
Stenman et al. (2016) [113]	Double-blind, randomized, parallel, placebo-controlled clinical trial	Healthy adults aged 18–65 years (n = 225); placebo (n = 56); LU (n = 53); B420 (n = 48); mix (n = 52)	Prebiotic treatment: dietary fiber Litesse^®^ Ultra polydextrose (LU) (12 g/day); probiotic treatment: B420 (10^10^ CFU/day); mix treatment: LU + B420	Microcrystalline cellulose placebo (12 g/day)	6 months	Probiotic and Mix treatment: <body fat, WC and food intake; no effects of prebiotic treatment.
Verhoel et al. (2011) [114]	Randomized, placebo-controlled, cross-over, double-blind clinical trial	Normal weight and overweight adults aged 20–60 years (n = 29)	Prebiotic-supplemented diet containing (1) 10 g FOS/day or (2) 16 g FOS/day	Placebo based on maltodextrin 16 g/day	13 days	>PYY in treatment (2); no effects on appetite, satiety, GLP-1 and energy intake
Whelan et al. (2006) [115]	Prospective, randomized, double-blind, cross-over trial	Healthy adults aged 28–30 years (n = 11)	Prebiotic-supplemented liquid enteral formula (18 g pea fiber + 10 g FOS/day)	Standard enteral formula (Nutren 1.0, Nestlé)	2 weeks	>Fullness and satiety

RCT: randomized clinical trials; BMI: body mass index; DRIs: dietary reference intakes; FOS: fructo-oligosaccharide; GLP-1: glucagon-like peptide-1; GLP-2: glucagon-like peptide-2; HOMA: homeostasis model assessment for insulin resistance; PYY: peptide YY; T2D: type 2 diabetes; WC: waist circumference.
